# Calcium Phosphate-Based Nanomaterials: Preparation, Multifunction, and Application for Bone Tissue Engineering

**DOI:** 10.3390/molecules28124790

**Published:** 2023-06-15

**Authors:** Xin Chen, Huizhang Li, Yinhua Ma, Yingying Jiang

**Affiliations:** 1 Department of Orthopedics, Jiading District Central Hospital Affiliated Shanghai University of Medicine & Health Sciences, Shanghai 201800, China; chenxin930@163.com (X.C.); eric164@126.com (H.L.); 2 Institute of Translational Medicine, Shanghai University, Shanghai 200444, China

**Keywords:** calcium phosphate, nanomaterials, bone tissue engineering, multifunction, drug delivery, bioimaging

## Abstract

Calcium phosphate is the main inorganic component of bone. Calcium phosphate-based biomaterials have demonstrated great potential in bone tissue engineering due to their superior biocompatibility, pH-responsive degradability, excellent osteoinductivity, and similar components to bone. Calcium phosphate nanomaterials have gained more and more attention for their enhanced bioactivity and better integration with host tissues. Additionally, they can also be easily functionalized with metal ions, bioactive molecules/proteins, as well as therapeutic drugs; thus, calcium phosphate-based biomaterials have been widely used in many other fields, such as drug delivery, cancer therapy, and as nanoprobes in bioimaging. Thus, the preparation methods of calcium phosphate nanomaterials were systematically reviewed, and the multifunction strategies of calcium phosphate-based biomaterials have also been comprehensively summarized. Finally, the applications and perspectives of functionalized calcium phosphate biomaterials in bone tissue engineering, including bone defect repair, bone regeneration, and drug delivery, were illustrated and discussed by presenting typical examples.

## 1. Introduction

Nowadays, over two million bone grafts are needed for bone defects [1,2], which are caused by trauma, infection, and tumors, since the size of the defect is far larger than the self-healing capability of bone tissue. Autologous bone grafts are still considered the gold standard to achieve bone defect repair [3,4]. Allografts also show excellent bioactivity, but the drawbacks are still obvious, including possible disease transmission, immune rejection, second injury, and donor site morbidity [5]. Thus, various bone substitutes have been made to solve the limitation of bone repair caused by the autologous bone, such as inorganic implants or organic implants. Usually, inorganic implants used for bone defects tend to be peri-implantitis [6], be non-degradable [7], and lack osteoinductivity [8], which bring about the continuous inhibition of bone regeneration and cause the absence of strong and effective mechanical support from newborn bone. Thus, patients may suffer a lot from default treatment.

Bone tissue engineering is a promising approach for the regeneration and repair of damaged bone tissues. Biomaterial-based approaches have emerged as an alternative to these traditional methods. Biomaterials can provide a scaffold for cell adhesion, proliferation, and differentiation, and they can also release growth factors and other bioactive molecules to promote bone regeneration. Biomaterials used for bone tissue engineering should have properties such as biocompatibility, biodegradability, osteoconductivity, and osteoinductivity. Biomaterials can be classified into the following four categories based on their composition: calcium phosphate-based biomaterials, metallic biomaterials, polymeric biomaterials, and composite biomaterials. Among these, calcium phosphate-based biomaterials have received the most attention due to their excellent biocompatibility, bioactivity, and similarity to the mineral component of bone [9,10].

Compared to conventional calcium phosphate-based graft materials, calcium phosphate nanomaterials offer several distinct advantages and show unique properties for bone tissue engineering, including enhanced bioactivity, tailored physical and chemical properties, controlled drug delivery, better integration with host tissue, and ease of fabrication and scalability. Specifically, these biomaterials have a high surface area to volume ratio, which provides more space for cell adhesion and proliferation and also promotes cell differentiation [11,12], as well as loading more therapeutic ingredients [13,14]. Additionally, the nano-sized particles can enhance the mechanical properties and show a profile of controlled drug release.

Thus, calcium phosphate nanomaterials have attracted more and more attention, and various preparation strategies have been developed to satisfy clinical requirements. The preparation methods include wet chemical precipitation, solvothermal synthesis, the sol-gel method, microwave-assisted method, sonochemical synthesis, the enzyme-assisted method, as well as spray drying and electrospinning. Among these, the precipitation method is the most commonly used method for the preparation of nano-calcium phosphate-based biomaterials, while the other methods also have their advantages, which are further discussed in the following section.

Furthermore, calcium phosphate-based nanomaterials can be tailored by adjusting their size, shape, and surface chemistry. Functionalized calcium phosphates are endowed with osteogenic properties, angiogenic properties, antimicrobial properties, bioimaging capabilities, and so on. Herein, calcium phosphate-based nanomaterials showed great potential in bone regeneration, antitumor therapy, and drug delivery. Thus, the review is intended to give a comprehensive summary of the preparation, multifunctionality, and application of nano calcium phosphate-based biomaterials for bone tissue engineering, and it also addresses the attention of readers and provides inspiration for the design of bioactive materials for bone tissue engineering.

## 2. Synthesis Strategies for Calcium Phosphate-Based Nanomaterials

Recently, lots of synthesis methods have been developed to prepare calcium phosphate nanomaterials, such as wet chemical precipitation, solvothermal synthesis, the sol-gel method, the microwave-assisted method, sonochemical synthesis, the enzyme-assisted method, as well as spray drying and electrospinning. This section gives a thorough introduction of the above-mentioned synthesis methods due to their unique advantages for the fabrication of calcium phosphate-based nanomaterials.

### 2.1. Wet Chemical Precipitation

**Figure 1:**
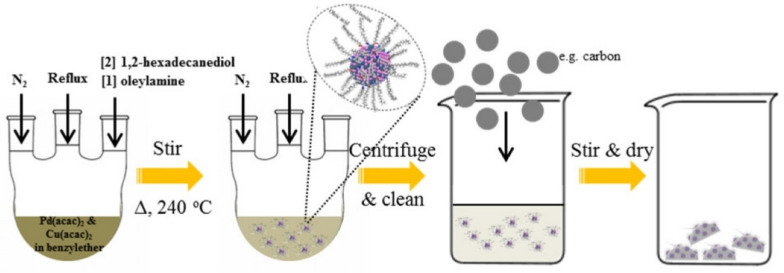
Schematic representation of wet chemical precipitation for the preparation of calcium phosphate-based biomaterial. Reprinted with permission from Ref. [24], Copyright 2019, American Chemical Society (Washington, DC, USA).

Wet chemical precipitation is thought to be the simplest method to prepare calcium phosphate nanomaterials, including amorphous calcium phosphate, which is the first solid phases of calcium phosphate, as well as hydroxyapatite, by providing mixed aqueous solutions of calcium ions and phosphate ions. As shown in Figure 1, the preparation of calcium phosphate-based nanomaterials usually involves the dispersion of the starting materials, typically calcium and phosphate precursors, in an aqueous solution, followed by a reaction between them to form a precipitate. The reaction is typically carried out under controlled conditions, such as fixed pH values and reaction times, to obtain the desired properties of the final biomaterials. After the reaction, the precipitate is washed with water or other solvents to remove impurities and obtain the final compound. The specific method used for the preparation of calcium phosphate-based nanomaterials may vary depending on the desired properties and application of the biomaterials.

The wet chemical precipitation method is a relatively simple and widely used method for the preparation of calcium phosphate-based nanomaterials [1]. However, there are some limitations and drawbacks to this method. For instance, the reaction time required for the precipitation process can be quite long, which may result in an incomplete reaction or the formation of impurities [15]. In addition, the resulting compound may not have optimal properties or functionality for certain applications. To overcome these limitations, researchers have explored various modification strategies. For example, the addition of dopants such as magnesium, cerium, and silicon can improve the functionality of the resulting biomaterials [16]. Surfactants, polymers, proteins, and bioactive molecules have been used to regulate the formation of CaP nanomaterials to regulate their components and phase. For example, in the presence of special medium, mimicking the situation of biomineralization in vivo, proteins and calcium ions can generate a kind of nanoparticle, which leads to a sustained release profile and increased bioactivity [17]. Additionally, microwaves and ultrasounds have also been introduced to improve reaction efficiency.

Moreover, precursor concentration, pH value, reaction temperature, mixing and stirring, and solvent influence the structure, morphology, and properties of nano calcium phosphate obtained through wet chemical precipitation [18,19]. Higher precursor concentrations can lead to larger particle sizes and increased crystallinity, while lower concentrations may result in smaller and more amorphous particles [20]. The optimization of precursor concentrations helps to achieve desired particle properties. The pH value of the reaction solution influences the solubility of calcium and phosphate ions and also affects the precipitation kinetics. Varying the pH can lead to different crystalline phases and morphologies of calcium phosphate nanoparticles, and it can also affect the subsequent implications for bioactivity and biocompatibility [21]. Higher temperatures during the precipitation process generally promote faster crystal growth, leading to larger particles with enhanced crystallinity. Conversely, lower temperatures tend to favor the formation of amorphous or poorly crystalline particles [22]. The method and intensity of mixing or stirring have an impact on particle size distribution, agglomeration, and homogeneity. Proper mixing promotes the uniform dispersion of precursors, resulting in more homogeneous nanoparticles. The solvent being in wet chemical precipitation can also affect the reaction kinetics [23], solubility of precursors, and crystal growth.

Overall, though the wet chemical precipitation method has its limitations, it remains an important and widely used method for the preparation of calcium phosphate-based nanomaterials. Researchers continue to explore and optimize this synthesis method to enrich the functionality of CaP nanomaterials for bone tissue engineering.

### 2.2. Solvothermal Synthesis

**Figure 2:**
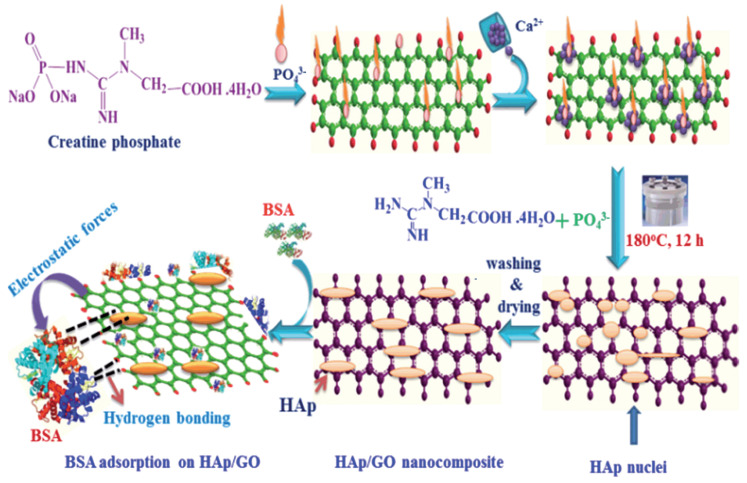
Schematic representation of solvothermal synthesis for the preparation of calcium phosphate-based biomaterial. Reprinted with permission from Ref. [31], Copyright 2016, Royal Society of Chemistry (London, UK).

The solvothermal synthesis method is a variation of the hydrothermal method, where organic solvents are used instead of water. This method allows for the use of higher temperatures and pressures, which can increase the solubility of the reactants and promote more rapid reaction rates (Figure 2) [25,26].

In a typical solvothermal synthesis procedure, the starting materials are mixed in a solvent using magnetic stirring. The mixture is then loaded into a sealed container, such as an autoclave, and heated to a high temperature for a fixed period of time. The pressure inside the container is also increased, which can further enhance the solubility of the reactants and accelerate the reaction. After the reaction is complete, the resulting product is typically washed with water or alcohol to remove any impurities or residual reactants. Finally, the product is vacuum-dried to remove any remaining solvent, and the final product can be obtained. Temperature and pressure, reaction time, precursor concentration, solvent, additives, and catalysts influence the structure, morphology, and properties of nano calcium phosphate obtained through solvothermal synthesis. Solvothermal synthesis involves heating the reaction mixture at elevated temperatures and pressures within a sealed autoclave. Temperature and pressure conditions have a significant effect on crystal growth, phase formation, and particle size distribution. Higher temperatures and pressures generally promote faster crystal growth, resulting in larger particles with enhanced crystallinity [27]. Longer reaction times allow for more extensive crystal growth, leading to larger particles, while prolonged reaction times may also result in increased agglomeration or phase transformation [28]. Higher precursor concentrations can lead to the formation of larger particles with increased crystallinity, but at excessively high concentrations, particle agglomeration may occur [29]. Different solvents have varying solubility properties, which affect the precursor dissolution and subsequent precipitation processes. Additional additives or catalysts can also be introduced in solvothermal synthesis to modify the particle characteristics [30]. For example, surfactants or templates can be employed to control particle size or morphology. Catalytic agents may be used to enhance the reaction rate or promote specific crystal phases.

The solvothermal synthesis method has been used to prepare a variety of calcium phosphate-based nanoparticles with controlled size, morphology, and composition. This method allows for greater control over the reaction conditions and can result in the production of biomaterials with improved properties and functionality compared to those obtained by other methods [31]. One of the main benefits of the solvothermal synthesis method is its ability to dissolve almost any material in the solvent, which can be heated and pressurized to its melting point. This allows for the synthesis of highly ordered nanostructures with controlled size, morphology, and composition. For example, the solvothermal method has been used to synthesize highly ordered arrays of microtubes made from a calcium chloride (CaCl_2_) powder precursor, which can form a block of three-dimensional structure. In addition, the solvothermal method has also been used to transform unstable di-tert-butyl phosphate into a stable precursor for calcium phosphate ceramic biomaterials using different calcium sources [32].

However, the solvothermal method does have some challenges; one of the major weaknesses is the difficulty in controlling the morphology and structure of the resulting nanostructures. This is due to multiple variables, such as the type, volume ratio, and concentration of the starting materials, as well as the reaction conditions, such as temperature and pressure [33]. Furthermore, the use of organic solvents can also pose some challenges, such as the need for appropriate safety precautions and the potential for toxicity [34]. Despite these challenges, the solvothermal method remains an important technique for the synthesis of CaP nanoparticles with tailored properties and functionality for bone tissue engineering and other applications.

### 2.3. Sol-Gel Method

**Figure 3:**
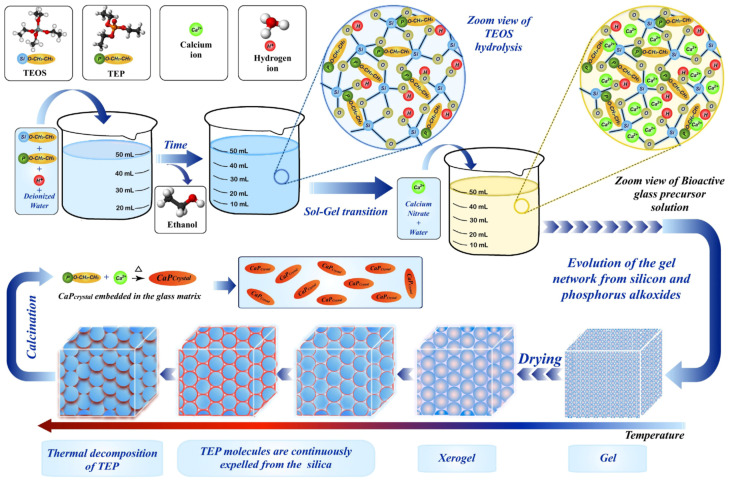
Schematic representation of the classical sol-gel procedure for the preparation of calcium phosphate-based bio-material. Reprinted with permission from Ref. [35], Copyright 2020, Elsevier (Amsterdam, The Netherlands).

As shown in Figure 3, the sol-gel method is another wet-chemical method for the synthesis of calcium phosphate-based nanoparticles. It is a relatively new procedure that does not require high temperature to help the compound reaction [35]. The sol-gel method typically involves several stages, including hydrolysis and polycondensation, gelation, drying, and crystallization. In the first step, inorganic materials, such as chlorides, nitrates, and sulfides, or metal alkoxides are dissolved in an acidic or alkaline solution and are stirred for a long time to obtain precursors. Metal alkoxides are then added to the precursors for polymerization, which leads to the development of a gel. Finally, the gel is converted into solid materials by freeze-drying or drying with high temperature [36,37] and can be used for coating scaffolds without any other drying.

Precursor composition, pH and solvent, hydrolysis and condensation ratios, drying methods, and annealing and heat treatment contribute to the regulation of the structure, morphology, and properties of nano calcium phosphate obtained through the sol-gel method [38]. Different precursors, such as inorganic salts or alkoxides, can lead to variations in the stoichiometry, crystallinity, and phase composition of the nanoparticles [39]. Varying the pH can affect the hydrolysis and condensation reactions, which, in turn, influence the particle size, surface charge, and morphology. The hydrolysis of the precursor and polycondensation of the resolved chemical products are the most significant steps in this synthesis [40]. Through this process, a wide range of diameters of nanoparticles can be synthesized by varying the ratio of materials [41]. These steps also affect the features of the final composites, such as specific surface area and microporosity, which can be influenced by using different reagents, various ratios of raw materials, or different temperature ranges [36,42,43]. Different drying techniques, such as freeze-drying or high-temperature drying, can lead to variations in the particle size distribution, agglomeration, and phase composition. Annealing can induce phase transformations, crystal growth, and improved crystallinity. The temperature and duration of heat treatment can be optimized to achieve desired properties, such as enhanced crystallinity, improved stability, and controlled release behavior.

Though there are still big challenges, the sol-gel method is still widely used due to its ability to produce CaP nanomaterials with high purity, good crystallinity, and controllable morphologies, making it suitable for various biomedical applications, including bone tissue engineering.

### 2.4. Microwave-Assisted Method

**Figure 4:**
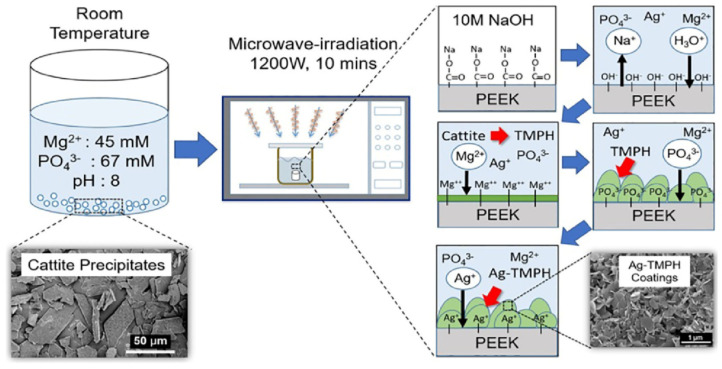
Schematic representation of the microwave-assisted procedure for the preparation of calcium phosphate-based biomaterials. Reprinted with permission from Ref. [45], Copyright 2020, Elsevier.

Microwave-assisted synthesis is a method of heating and accelerating the chemical reaction between materials to obtain CaP particles [44,45]. As shown in Figure 4 [45], the process involves dissolving soluble materials in a solvent, adjusting the pH value of the mixture, subjecting the mixture to microwave irradiation at a fixed temperature and time, and finally isolating the product through centrifugation, cleaning, and drying [46,47].

Power and duration, solvent and pH, precursor concentration and ratio, additives and surfactants, and cooling rate influence the formation of CaP nanoparticles [48]. Higher power and longer durations can lead to increased heating and higher reaction temperatures, affecting the nucleation, growth, and crystallinity of the nano calcium phosphate particles [49]. Different solvents and pH conditions can affect the particle size, morphology, and phase composition of the nano calcium phosphate [17]. Varying the precursor concentrations and ratios can affect the nucleation and growth kinetics, leading to variations in particle size, crystallinity, and surface properties [50]. These additives can act as nucleation promoters, growth modifiers, or surface modifiers, resulting in controlled particle formation, improved dispersibility, and enhanced stability. Rapid cooling can lead to the formation of amorphous or partially crystalline phases, while slower cooling rates may promote the growth of larger crystals [51]. The cooling rate can influence the phase composition, crystallinity, and surface properties of the synthesized nanoparticles. Thus, the structure, morphology, phase, as well as particle size can be regulated via the parameters.

Microwave-assisted synthesis has some advantages, such as high energy efficiency, shorter synthesis time, as well as consuming few samples [39], which make it a promising method for the laboratory. However, the high heat generated can also lead to some disadvantages, such as the deterioration of the sample constituents and the high cost of industry-level equipment maintenance. It is important to consider both the advantages and disadvantages of this method before using it in research or industrial applications.

### 2.5. Sonochemical Synthesis

**Figure 5:**
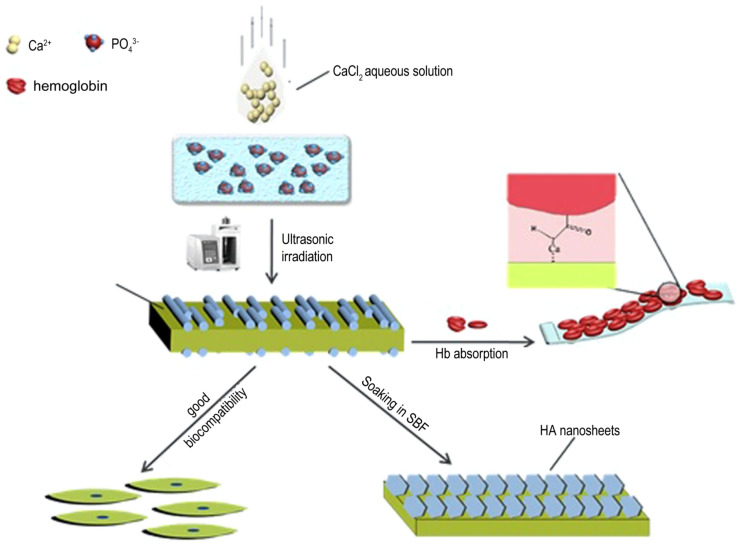
Schematic representation of the sonochemical synthesis for the preparation of calcium phosphate-based biomaterials. Reprinted with permission from Ref. [52], Copyright 2018, Nature Publishing Group (Berlin, Germany).

As shown in Figure 5, sonochemical synthesis has the potential to offer several advantages over other synthesis methods, such as being safer and more environmentally friendly, as it does not require the use of toxic solvents [52]. This method also has the advantage of the reduction in reaction time, the ability to control particle size, and the production of highly homogeneous materials. The method can also be carried out at relatively low temperatures and does not require high-pressure conditions, which reduces the energy consumption and cost of the process. Additionally, sonochemistry can be used to modify the surface properties of materials, such as increasing their porosity, improving their biocompatibility, and enhancing their drug delivery capabilities. Additionally, the cavitation effect can lead to smaller and more uniform particle size, which can increase the protein absorption of the nanoparticles [53]. Herein, ultrasonic power and frequency, [54] reactant concentration, [55] pH and temperature, the addition of surfactants and additives, and reaction time [56] all influence the formation of calcium phosphate nanoparticles via sonochemical synthesis.

Further research is needed to fully understand the effects of sonochemical synthesis on the properties of calcium phosphate-based nanoparticles, including their crystallinity and morphology [56]. With continued exploration and the refinement of this method, it may prove to be a valuable approach for the synthesis of high-quality calcium phosphate-based materials with a wide range of potential applications. The main disadvantage of sonochemistry is that it is difficult to scale up to industrial-level production due to the limitations of the equipment and the variability in the cavitation effect.

### 2.6. Enzyme-Assisted Method

**Figure 6:**
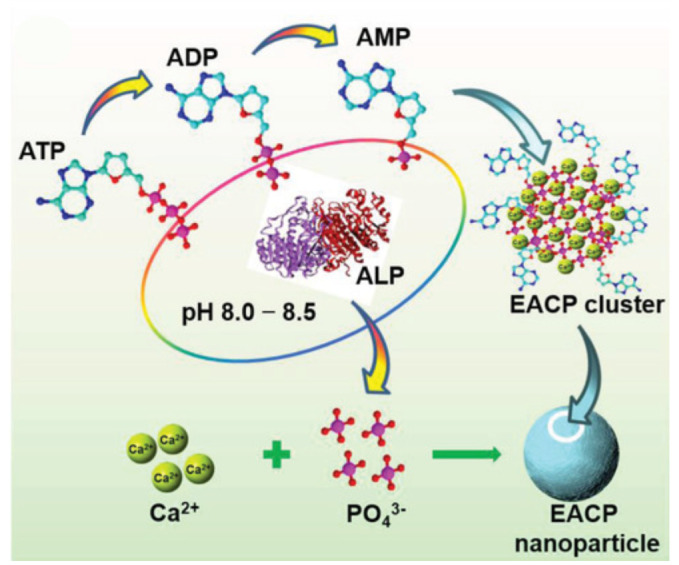
Schematic representation of the enzyme-assisted method for the preparation of organic-inorganic nanocomposites. Reprinted with permission from Ref. [58], Copyright 2018, Wiley (Hoboken, NJ, USA).

There is also research that shows that calcium phosphate-based nanoparticles, obtained in the enzymatic approach, have strong bioactivity in vivo/vitro experiments due to functional enzymes in promoting biomineralization [57]. As shown in Figure 6, Jiang et al. developed bioactive minerals via the enzymatic reaction based on the use of alkaline phosphatase (ALP) to catalyze the hydrolysis of ATP molecules and the release of phosphate ions combined with the unconstrained calcium ions in the precursor solutions. Thereafter, the organic-inorganic composites containing amorphous calcium phosphate, ATP, ADP, and AMP were obtained, and the composites showed superior biocompatibility, enhanced osteogenic differentiation, and reduced repair time for bone regeneration [58]. Additionally, the control of rate of deposition and final coating morphology can be reached by adjusting the value of the pH, the concentration of the enzyme, the ratio of calcium and phosphate ions, and the time of reaction [59].

Enzymatic methods offer several advantages in the synthesis of calcium phosphate-based biomaterials. Firstly, they are mild and environmentally friendly because the reaction is performed under mild conditions without the use of harsh chemicals or high temperatures [60]. Secondly, the obtained materials are usually of highly purity, which is essential for their biological activity. Thirdly, enzymatic methods have the potential to mimic the biomineralization processes that occur in living organisms, leading to the synthesis of biomaterials with unique properties that are not possible to achieve via other methods [57]. The enzymatic synthesis method also has some drawbacks. The enzymatic reaction is often slow and requires longer reaction times than other methods. Additionally, the enzyme used in the process can be expensive and can affect the final product yield.

### 2.7. Spray Drying and Electrospinning

Spray drying and electrospinning are two other methods used for the preparation of calcium phosphate nanomaterials. In the spray drying method, a solution containing the calcium and phosphate precursors is sprayed into a hot air stream, resulting in the formation of nanoparticles. In addition, the spray process can also be applied to the preparation of calcium phosphate coating [61]. In the electrospinning method, a polymer solution containing calcium and phosphate precursors is used to form nanofibers, which are then calcined to obtain the desired calcium phosphate nanomaterials.

Additionally, plasma spraying can also be used for the preparation of calcium phosphate coating, which can effectively prevent the corrosion and loosening of metal implants in clinic. Plasma spraying requires a high cost; therefore, the application is limited. Liu et al. developed a double-layer calcium phosphate-sandwiched siloxane composite coating that can decrease the cost by using the chemical conversion method and the sequential mineralization method to promote the loading of Ca and P elements with the coating so that this magnesium alloy with the composite coating can achieve good mineralization ability and biocompatibility in vivo [62]. Moreover, calcium phosphate coatings can also be obtained via sequential mineralization in SBF [63] or electrochemical-assisted deposition [64].

For spray drying and electrospinning, the concentration of the calcium and phosphate precursors in the spray drying and electrospinning solutions affects the viscosity and surface tension of the solution. Higher concentrations typically result in increased particle size and improved crystallinity. Different solvents have varying evaporation rates and compatibility with the precursors, [65] leading to differences in particle size, porosity, and surface characteristics. Higher airflow rates and temperatures generally result in smaller particle sizes and improved crystallinity. Optimal feed rates ensure uniform particle distribution [66]. Different polymers have varying viscosities, conductivity, and compatibility with the precursors, leading to differences in fiber morphology, diameter, and mechanical properties [67]. Post-treatment techniques, such as heat treatment or cross-linking, can influence the crystallinity, porosity, and mechanical strength of the nanoparticles.

As described above, each synthesis method has its own unique advantages and limitations for the preparation of calcium phosphate nanomaterials, and a summary is given in Table 1. Proper synthesis methods can be employed for preparing calcium phosphate nanomaterials with desired properties.

## 3. Multifunctional Properties of Calcium Phosphate-Based Nanomaterials

The various synthesis methods contribute to the diversity of calcium phosphate-based nanoparticles. Herein, calcium phosphate-based nanomaterials have favorable properties, involving biocompatibility, biodegradability, and the fact that calcium is a critical mineral composition of human bones and teeth [85], and calcium phosphate-based nanomaterials can also decompose in an aqueous solution with the pH value in human internal environment [86]. Thus, it can be used as a great carrier to load drugs and can protect them in blood [87], leading to controllable release in the human internal situation.

Moreover, large specific surface areas of calcium phosphate-based nanomaterials lead to loading more therapeutic drugs, increasing the concentration of the drug around the targeted sites to achieve efficient bone healing [88,89]. Based on this reason, Kans et al. developed CaP-based coatings with interconnected pores via ultrasound-assisted microarc oxidation method. The porous structure of the coatings contributes to the improvement of the vancomycin loading capacity to achieve more effective antibacterial properties. Additionally, by taking advantage of the electrostatic interaction between Ca^2+^ ions, on the surface of the CaP, and COO^-^ in bovine serum albumin (BSA), Kanupriya et al. found that anhydrous dicalcium phosphate synthesized via rapid microwave-assisted synthesis had higher porosity (77.8 m^2^/g), providing more binding sites for BSA. The porous anhydrous dicalcium phosphate showed about a 59 wt% loading capacity of BSA, which is much higher than that of hydroxyapatite with a lower specific surface area (16.1 m^2^/g) [90].

**Figure 7:**
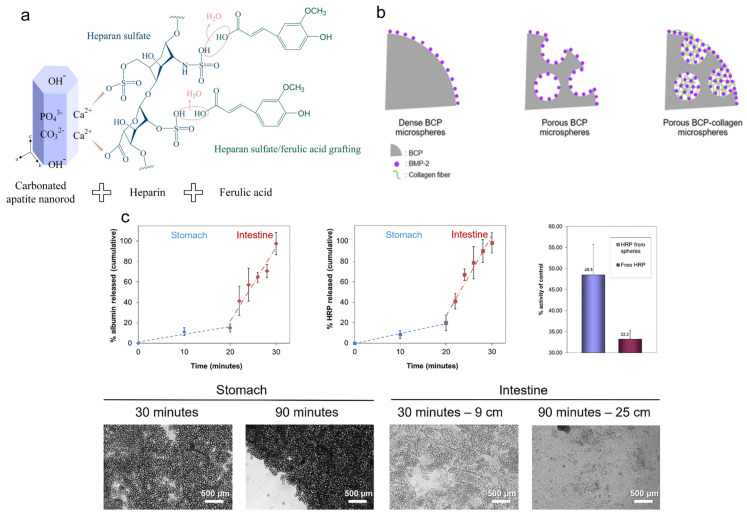
Strategies for the component engineering of nano-calcium phosphate-based biomaterials. (**a**) Calcium phosphate-based biomaterial coated with heparin to crosslink with ferulic acid; reprinted with permission from Ref. [97], Copyright 2021, MDPI (Basel, Switzerland). (**b**) The crosslinked porous biphasic calcium phosphate loading more BMP-2; reprinted with permission from Ref. [95]. (**c**) The structure of the calcium phosphate-based biomaterial influenced by the pH value, leading to the drug release; reprinted with permission from Ref. [98], Copyright 2019, Elsevier.

As shown above, calcium phosphates have the ability to incorporate biomolecules, inside or on the surface, to reach the target sites and deliver therapeutic compounds. In addition, some biomolecules can combine with inorganic structures to repair bone defects by suppressing the acute inflammatory response [91,92] or various active ingredients, promoting the formation of new blood vessels, inhibiting the fusion and migration of osteoclasts [93] or directly encouraging osteogenic differentiation [94,95,96]. As shown in Figure 7a, Kai-Chi et al. developed uniform carbonate-based apatite nanorods, which upregulate cellular attachment and mineralization, by combining ferulic acid through the regulation of heparin [97]. Additionally, many growth factors show various functions to control the evolution, differentiation, and metabolism of stem cells in the process of bone regeneration, including platelet-derived growth factor (PDGF), transforming growth factor beta1 (TGFbeta1), vascular endothelial growth factor (VEGF), basic fibroblast growth factor (bFGF), hepatocyte growth factor (HGF), epidermal growth factor (EGF), insulin-like growth factor-1 (IGF-1), and so on. Bone-morphogenetic proteins (BMP) show especially promising results when used in bone regeneration. As shown in Figure 7b, Seong et al. combined BMP-2 with the crosslinked porous biphasic calcium phosphate, invented by applying a water-in-oil emulsion technique with camphene as a pore generator, to achieve high loading efficiency and constant release [95]. In another study shown in Figure 7c, there is a phenomenon that the calcium pyrophosphate can keep the shape of nanofiber microspheres in the presence of a low pH value (pH~4), consisting of a stomach environment, and can reorganize into fiber and even into mineral plates in the intestinal environment (pH~7), which means that the large specific surface area of 65 m^2^/g turns into only 1.5 m^2^/g; thus, the loaded protein can keep its high activity when exposed to the stomach environment. Subsequently, the loaded proteins would be released in the intestinal environment, which leads to targeted enteric delivery [98].

Stem cells, such as hBMSCs [10], hESC-MSCs [99], and hiPSC-MSCAs [100], play a critical role in the process of bone regeneration. Several studies have investigated that calcium-based materials can load these cells [101], and the interaction between nanostructures and cells contributes to bone healing [102,103]. Calcium phosphate bone cement (CPC) was compounded with bone marrow mesenchymal stem cells (BMSCs) and was condensed in alginate chitosan alginate (ACA) microcapsules. This can protect cells from the negative influence of the CPC setting reaction and effectively form macropores in the composite to help CPC display better bone regeneration functions in the performance of ectopic osteogenesis and high effective degradability in vivo compared to the CPC with the addition of pure microcapsules or cell microcapsules [103]. Calcium phosphates, which are osteoconductive, injectable, and moldable, can provide strong sites for the proliferation and differentiation of cells and can promote bone healing.

**Figure 8:**
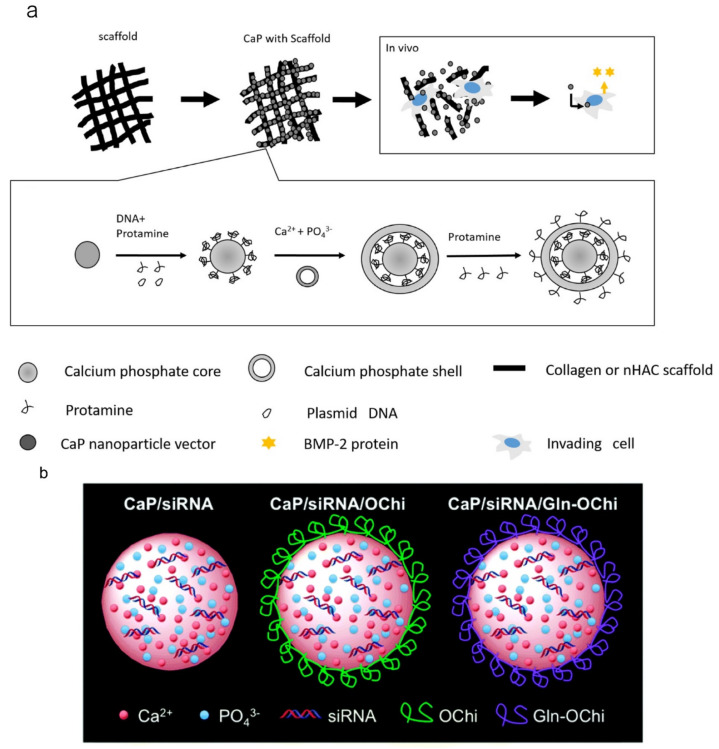
Strategies for calcium phosphate-based material loading genes. (**a**) Calcium phosphate-loading DNA fixed in a scaffold to promote BMP-2 protein; reprinted with permission from Ref. [96], Copyright 2018, Elsevier. (**b**) The nucleic acid-loaded nanoparticles, combining nucleic acid molecule-loaded CaP nanoparticles with a Gln-Ochi chitosan coating to avoid aggregating; reprinted with permission from Ref. [108], Copyright 2015, Royal Society of Chemistry.

Gene therapy is a potential and new approach to achieve highly efficient bone regeneration [104]. Calcium phosphate nanomaterials can be used as carriers for RNA [85,105] or DNA [106,107]. BMP-2 promotes bone regeneration effectively, and a high dosage of it can be loaded on the calcium phosphate nanocarriers to achieve the satisfied release of the growth factor during the stage of bone regeneration. However, there is difficulty in achieving the controlled release of growth factors during bone regeneration. A fast degradation rate leads to the release of BMP-2 at a high level, as well as bone or tooth resorption. To solve this problem, achieving larger, longer, and a more gradual release of BMP-2, as shown in Figure 8a, Tenkumo et al. designed a multi-shell calcium phosphate nanoparticle as a carrier with BMP-2-encoding-plasmid DNA. Thereafter, the drug delivery system was incorporated with a nanohydroxyapatite-collagen scaffold to promote gene transfection successfully [96]. The efficient cellular uptake of nanoparticle-loaded nucleic acid molecules has a huge influence on the efficiency of bone regeneration. In Figure 8b, it is shown that Bogyu Choi et al. found that nucleic acid-loaded CaP nanoparticles with a Gln-Ochi chitosan coating can protect these particles from forming large aggregates due to electrostatic repulsion and can achieve higher cellular uptake to promote bone tissue regeneration.

**Figure 9:**
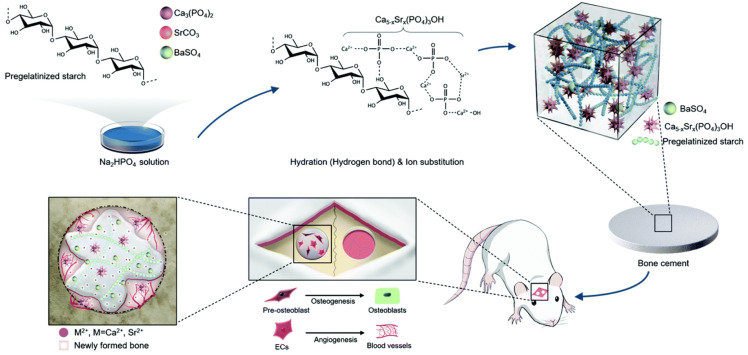
Strategies for mixing metal ions with calcium phosphate. Calcium phosphate cement mixed with Ba/Sr ions to achieve bone regeneration. Reprinted with permission from Ref. [114], Copyright 2021, Royal Society of Chemistry.

Metal ions can also be integrated within calcium phosphate-based nanomaterials, regulating gene expression and promoting bone repair [109,110,111,112,113]. As shown in Figure 9, Wu et al. introduced strontium ions, which came from SrCO_3_, into the modified calcium phosphate cement, composed of 20 wt% pregelatinized starch and 10 wt% BaSO_4_, to improve the flexibility, uniformity, and radiopacity performance of the cement. A total of 0.25 M of the Na_2_HPO_4_ solution acted as the setting liquid. This mixture performed well in a calvarial defect model of a rat and showed suitable mechanical strength [114].

## 4. Applications of Calcium Phosphate-Based Nanomaterials in Bone Tissue Engineering

**Figure 10:**
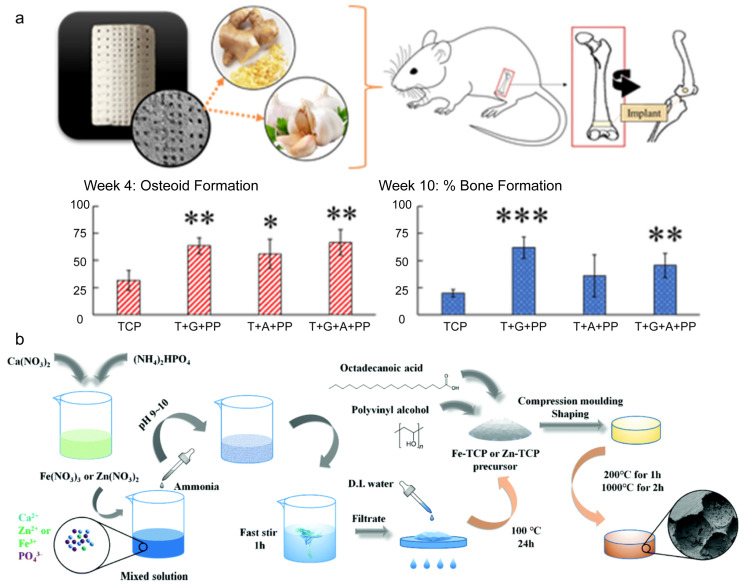
Calcium phosphate composite materials with different properties for bone repair. (**a**) Calcium phosphate scaffolds loaded with ginger and garlic extracts can improve bone regeneration and osteogenic differentiation (* *p* < 0.05; ** 0.05 < *p* < 0.01; and *** *p* < 0.01); reprinted with permission from Ref. [116], Copyright 2022, American Chemical Society. (**b**) Calcium phosphate materials were combined with Zn/Fe ions to turn micropores into a dispersed solid; reprinted with permission from Ref. [117], Copyright 2019, Royal Society of Chemistry.

Calcium phosphate-based nanomaterials show superior biocompatibility, show osteoinductivity, and have high surface area and porosity. Thus, they have been widely used for bone regeneration [115]. Additionally, calcium phosphate-based nanomaterials can be doped with special compounds to promote bone repair directly. Susmita et al. designed 3D calcium phosphate scaffolds, resembling the structure of cortical and cancellous bones, to provide a stronger, denser structure to develop compressive strengths of the scaffold and maintain the central interconnected porosity for cell proliferation and vascularization. The scaffold was incorporated with natural compounds from ginger root and garlic powder, which can enhance bone healing when used in vivo, and improved the bioavailability of these medicinal compounds [116] (Figure 10a). Furthermore, many metal elements also have various influences on the process of bone regeneration, such as silicon, magnesium, cerium, and so on. Based on this theory, Fe and Zn were combined with tricalcium phosphate (TCP) via co-deposition because these two elements can improve new blood vessel formation, which can significantly promote bone regeneration; then, the Fe/Zn-modified TCP powders were turned into a solid, and micropores were formed inside at room temperature. The composites achieved the sustained release of metal (Ca^2+^, Zn^2+^ and Fe^3+^) ions, which can promote the process of bone repair [117] (Figure 10b). Additionally, because of the low mechanical strength, brittleness, and quick resorption rate, CaP-based scaffolds need to be reinforced with mechanical strength and be improved regarding bioactivity. It is reported that bioceramic powders (hydroxyapatite and β-tricalcium phosphate) have been embedded in mixed solutions of chitosan and silk fibroin to improve both the mechanical properties and the differentiation of mesenchymal stem cells since chitosan and silk fibroin have shown better bioactivity in supporting the growth and differentiation of BMSCs. Additionally, the polymer solution can be used as the bioink of robocasting equipment to print 3D scaffolds with fixed system parameters at room temperature, and this scaffold achieved great performance regarding mechanical properties and biocompatibility [118]. Calcium phosphate-based materials can also be used as carriers to load cells or growth factors, which can improve effective therapeutic ingredients in bone defect areas, can keep the sustained release of these factors, and can provide enough room for osteoblasts and new blood vessels [119]. Li et al. reported that bone marrow mesenchymal stem cells and platelet-rich plasma combined with a calcium phosphate cement scaffold achieved more newly formed bone areas than normal calcium phosphate cement scaffolds in an in vivo experiment [120].

**Figure 11:**
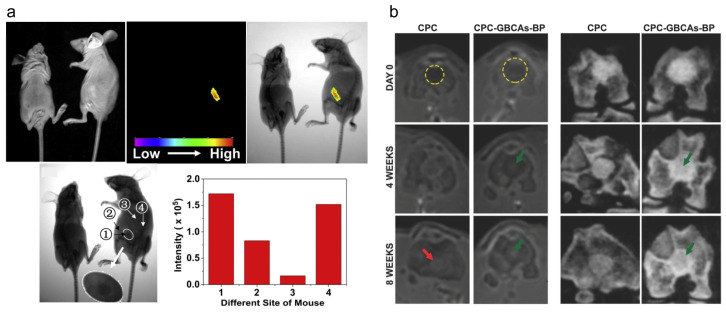
Multi-application in the imaging area. (**a**) Calcium phosphate-based materials used in bioimaging; reprinted with permission from Ref. [124], Copyright 2012, Elsevier. (**b**) Simple flow chart of calcium phosphate composite gadolinium and for CT and MRI bone defect repair evaluation. Green arrows indicate the bright area which appears in the middle of the implant on the MRI acquisitions after 4 weeks from the surgery. Red arrow displays the calcium phosphate cements that become indistinguishable from the surrounding bone after 8 weeks from implantation in vivo; reprinted with permission from Ref. [126], Copyright 2018, Wiley.

Recently, near-infrared-emitting persistent luminescence nanoparticles have been used as an optical probe for bioimaging and biosensing [121,122]. Though they show low absorptivity in deep-tissue imaging, achieving stable in vivo luminescence is still a great challenge [123]. Chen et al. developed Eu^3+^/Gd^3+^ ion-dual-doped CaP nanoparticles via co-precipitation using block copolymer polylactide-block-monomethoxy as a template. The nanoparticles showed prolonged stability (more than 80 days), improved the T1-weighted MRI signal intensity, and decreased T2-weighted MRI signal intensity, which resulted in darker images for the better observation of changes in local areas in vivo [124] (Figure 11a). Additionally, to monitor the in vivo performance of calcium phosphate cements, which show a high structural similarity to bone [125], calcium phosphate cements linked with gadolinium (III) by bisphosphonates were developed, and they showed shortened T1 relaxation times of the water in tissues and achieved signal enhancement in a T1-weighted MRI. In this way, the highly accurate evaluation of the implant shape both in MRI and CT for a prolonged time period (about 8 weeks) was achieved [126] (Figure 11b).

**Figure 12:**
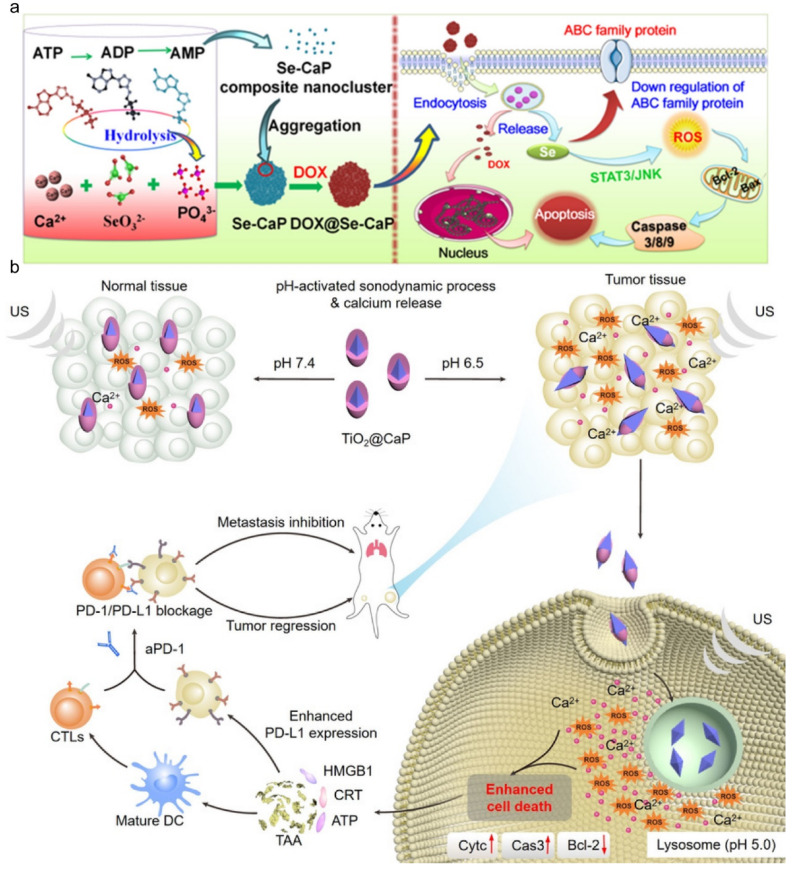
Strategies for calcium phosphate-based materials to kill cancer cells. (**a**) Reversal of the multidrug resistance of tumors by combining calcium phosphate with Se element; reprinted with permission from Ref. [130], Copyright 2020, Elsevier. (**b**) Construction of calcium phosphate and TiO_2_ to promote cancer therapy; reprinted with permission from Ref. [142], Copyright 2021, Wiley.

Calcium phosphate-based nanoparticles are also desirable materials for drug delivery [127]. Doxorubicin could activate the immunogenic cell death of tumor cells through an apoptosis pathway, including reactive oxygen species, which could lead to DNA cleavage and mitochondrial dysfunction [128], while the multidrug resistance of cancers may result in poor prognosis [129]. To address this issue, a selenium-doped CaP, serving as the doxorubicin carrier, was designed for reversing the multidrug resistance of tumors. Se-doped CaP is pH-sensitive and dox can be released specifically in the tumor sites due to the acid microenvironment. Thus, the uptake of anticancer drugs of tumor cells was raised, and Se ions released could decrease reactive oxygen species of tumor cells and the expression of ATP-binding cassette transporters, which reverse multidrug resistance [130] (Figure 12a). The surgery of bone tumors causes large bone defects, and calcium phosphate bone pastes have been widely used as bone substitutes in clinics to replace allogenous bone, providing strong and stable support for the defect sites without internal fixation [131,132], for many years, and the result of anticancer is also well described in the long run [133,134]. Furthermore, there is the theory that calcium ions play a vital role in tumor necrosis by regulating the stability of the intracellular concentration of calcium ions [135,136,137], and a high concentration of calcium in the microenvironment of the tumor can encourage tumor calcification [138,139]. Based on this phenomenon, calcium phosphate can combine with some special materials, such as ferritin, which is a promising load for targeted delivery, to protect ferritin from interception to achieve the function of targeted delivery to the tumor effectively, and the shell of calcium phosphate not only counterbalances the acidic microenvironment around tumors but also accelerates the immunomodulation and calcification of cancers [140,141]. CaP nanoparticles can also be coated to camouflage, which may result in intracellular calcium overload and induce apoptosis. For example, TiO_2_-coated CaP can elevate the generation of reactive oxygen species, due to the demonstrative property of inorganic sonosensitizers, and can provide calcium ions due to the acidic microenvironment of the tumor (Figure 12b) [142,143,144].

## 5. Conclusions and Perspectives

Nano-calcium phosphate-based biomaterials have shown great potential in bone tissue engineering due to their unique properties, including their small size, high surface area to volume ratio, and multifunctionality. Various preparation methods, including sol-gel, hydrothermal synthesis, spray drying, and electrospinning, have been used to obtain these biomaterials with controlled size, morphology, and composition.

The multifunctionality of nano-calcium phosphate-based biomaterials is attributed to their abilities to enhance the osteogenic differentiation and mineralization of mesenchymal stem cells, to promote angiogenesis, and to exhibit antimicrobial properties. These properties make them attractive for various applications in bone tissue engineering, including bone defect repair, bioimaging, and anticancer applications.

The preparation, multifunctionality, and application of nano-calcium phosphate-based biomaterials for bone tissue engineering have been extensively studied and reviewed in the literature. These biomaterials have shown great potential in the regeneration and repair of bone tissues and can provide a scaffold for the adhesion, proliferation, and differentiation of stem cells. Further research is needed to optimize the preparation methods, functionalization, and characterization of these biomaterials to improve their performance and clinical outcomes. Nonetheless, these biomaterials represent a promising avenue for the development of effective and safe bone tissue engineering therapies.

The future exploration of calcium phosphate-based nanomaterials could be based on the following points: more effective binding of active substances; more effective targeting methods to achieve more effective treatment goals in local areas; and the interaction mechanism between calcium phosphate and substances. Calcium phosphate binds to the active ingredient and can protect the bioactivity of the component from being affected to maximize the function of the ingredient, such as promoting bone regeneration and anti-cancer properties. In addition, effective targeting strategies can deliver calcium phosphate-loaded components to the relevant areas so that the local area has a high concentration of therapeutic components, which minimizes the harm caused by therapeutic components. In order to achieve the above objectives, it is necessary to further explore the interaction mechanism between calcium phosphate and various substances to achieve the stable synthesis of calcium phosphate materials with related functions and even commercial production into clinical applications. Thus, it is conducive to the stable output of bioactive calcium phosphate material for large-scale production and application in practice.

Thus far, based on the consideration of biosafety, only a few calcium phosphate-based nanoparticles have been applied in clinics. Therefore, we hope that this review inspires readers to obtain more ideas for the design of bone repair materials.

## Figures and Tables

**Table 1:** A summary of synthesis methods for the preparation of calcium phosphate-based nanomaterials.

Synthesis Methods	Advantages	Disadvantages
Wet chemical precipitation	Simple, efficient [68] Easy control of the reaction system environment [19]	Needs a long reaction time [19] Difficult to purify composites [69]
Solvothermal synthesis	Short reaction time [28] Simple operation process [29]	Security risks in the reaction process [70] Hard to control materials’ properties [71] More energy consumption [72]
Sol-gel method	Simple synthesis process [73] Excellent calcium phosphate crystallization [74]	The physicochemical properties of the product are uncontrollable [75] Mixed with impurities [76]
Microwave-assisted method	Short reaction time [51] Less raw material consumption [48]	Raw materials require full dissolution [45] Limited application range [17] High equipment maintenance costs [77]
Sonochemical synthesis	Less cost of the process [55] The cavitation effect [52]	Few studies on the preparation of calcium phosphate materials [78] Hard to industrialize [53]
Enzyme-assisted method	Environmentally friendly [79] The reaction conditions are controllable [58]	Longer reaction time [80] Whole process monitoring [81]
Spray drying and electrospinning	Machine parameters control product structure [82]	High equipment costs [83] Difficult preparation of precursor solution [84]

## Data Availability

Not applicable.
